# Arterial Spin Labeling Imaging Characteristics of Anti-leucine-rich Glioma-Inactivated 1 Encephalitis: A Qualitative and Quantitative Analysis

**DOI:** 10.3389/fneur.2022.850029

**Published:** 2022-07-28

**Authors:** Vivek Srikar Yedavalli, Omar Hamam, Mona Bahouth, Victor Cruz Urrutia, Amara Ahmed, Hanzhang Lu, Craig Jones, Licia Pacheco Luna, Haris Iqbal Sair, Bryan Lanzman

**Affiliations:** ^1^Russell H. Morgan Department of Radiology and Radiological Sciences, Johns Hopkins School of Medicine, Baltimore, MD, United States; ^2^Florida State University School of Medicine, Tallahassee, FL, United States

**Keywords:** Arterial Spin Label (ASL) MRI, stroke mimics, encephalitis, MRI, perfusion imaging

## Abstract

**Background and Significance:**

Autoimmune encephalitis (AE) is a rare group of diseases that can present with stroke-like symptoms. Anti-leucine-rich glioma inactivated 1 (LGI1) encephalitis is an AE subtype that is infrequently associated with neoplasms and highly responsive to prompt immunotherapy treatment. Therefore, accurate diagnosis of LGI1 AE is essential in timely patient management. Neuroimaging plays a critical role in evaluating stroke and stroke mimics such as AE. Arterial Spin Labeling (ASL) is an MRI perfusion modality that measures cerebral blood flow (CBF) and is increasingly used in everyday clinical practice for stroke and stroke mimic assessment as a non-contrast sequence. Our goal in this preliminary study is to demonstrate the added value of ASL in detecting LGI1 AE for prompt diagnosis and treatment.

**Methods:**

In this retrospective single center study, we identified six patients with seropositive LGI1 AE who underwent baseline MRI with single delay 3D pseudocontinuous ASL (pCASL), including five males and one female between ages 28 and 76 years, with mean age of 55 years. Two neuroradiologists qualitatively interpreted the ASL images by visual inspection of CBF using a two-point scale (increased, decreased) when compared to both the ipsilateral and contralateral unaffected temporal and non-temporal cortex. The primary measures on baseline ASL evaluation were a) presence of ASL signal abnormality, b) if present, signal characterization based on the two-point scale, c) territorial vascular distribution, d) localization, and e) laterality. Quantitative assessment was also performed on postprocessed pCASL cerebral blood flow (CBF) maps. The obtained CBF values were then compared between the affected temporal cortex and each of the unaffected ipsilateral parietal, contralateral temporal, and contralateral parietal cortices.

**Results:**

On consensus qualitative assessment, all six patients demonstrated ASL hyperperfusion and corresponding FLAIR hyperintensity in the hippocampus and/or amygdala in a non-territorial distribution (6/6, 100%). The ASL hyperperfusion was found in the right hippocampus or amygdala in 5/6 (83%) of cases. Four of the six patients underwent initial follow-up imaging where all four showed resolution of the initial ASL hyperperfusion. In the same study on structural imaging, all four patients were also diagnosed with mesial temporal sclerosis (MTS). Quantitative assessment was separately performed and demonstrated markedly increased CBF values in the affected temporal cortex (mean, 111.2 ml/min/100 g) compared to the unaffected ipsilateral parietal cortex (mean, 49 ml/min/100 g), contralateral temporal cortex (mean, 58.2 ml/min/100 g), and contralateral parietal cortex (mean, 52.2 ml/min/100 g).

**Discussion:**

In this preliminary study of six patients, we demonstrate an ASL hyperperfusion pattern, with a possible predilection for the right mesial temporal lobe on both qualitative and quantitative assessments in patients with seropositive LGI1. Larger scale studies are necessary to further characterize the strength of these associations.

## Introduction

Autoimmune encephalitis (AE) is a family of immune-mediated diseases, which can present with generalized stroke-like symptoms, such as seizures and cognitive deficits, making prompt and accurate diagnosis challenging (1, 2). Autoimmune encephalitis is subdivided into two groups based on the location of the autoantibody target; Group I has intracellular targets and is more closely associated with an underlying malignancy, while Group II autoantibodies bind to cell surface antigens and are less likely to be paraneoplastic (2). Group II AE is also generally more responsive to treatment compared to Group I. Leucine-rich glioma-inactivated 1 (LGI1) AE is a Group II AE and second most common AE subtype (3), with an annual incidence of 0.83 per million people (1). LGI1 is seen in 11.2% of all AE cases (4) and classically presents with faciobrachial dystonic seizures (FBDS) in addition to cognitive deficits (4). Unlike some AE forms (typically Group II), LGI1 AE is not commonly associated with neoplasms and responds well to immunotherapy, where studies have shown that 67% of patients can achieve favorable outcomes (1). The timing of the immunotherapy is also important. When there is high clinical suspicion for AE and confirmatory antibody testing is not readily available, expeditious administration of immunotherapy is associated with better outcomes (5). Prompt and accurate diagnosis of AE and its subtypes is essential to begin immunotherapy earlier and potentially decrease likelihood of complications, such as mesial temporal sclerosis (MTE) (6).

In conjunction with the clinical presentation, physiological neuroimaging plays an important role in diagnosing AE subtypes. Fluorodeoxyglucose positron emission tomography (PET) has been shown to aid in differentiating AE subtypes based on spatial differences in metabolic activity. For example, anti-methyl-d-aspartate receptor encephalitis (anti-NMDA) demonstrates frontotemporal hypermetabolism with occipital hypometabolism (5). In contrast, contactin-associated protein-2 AE (CASPR-2) and LGI1 have both been associated with mesial temporal hypermetabolism (7).

Arterial Spin Labeling (ASL) is a non-contrast MRI perfusion technique that is able to measure cerebral blood flow (CBF) (8). It has been increasingly utilized in clinical practice due to its ability to provide physiological assessment. It has shown utility in stroke and stroke-mimic detection in particular (9–14). PET and ASL have shown moderate to strong concordance in detecting a number of pathologies, including mesial temporal lobe epilepsy (15). As with PET, reports have suggested that ASL can aid in detecting AE subtypes, such as anti-NMDA (16) and anti-glutamic acid decarboxylase (ani-GAD) (17) when preceding laboratory diagnosis. A recent case report with two patients has demonstrated strong concordance between ASL and PET in detecting LGI1, specifically on qualitative visual inspection (18). Nevertheless, the role of ASL in detecting AE subtypes and LGI1 in particular is vastly underexplored. Therefore, the objective of this study is to characterize the ASL imaging findings in seropositive LGI1 AE.

## Materials and Methods

We retrospectively performed an imaging and chart review of six consecutive patients who were diagnosed with AE based on MRI imaging characteristics and given symptomatology from December 2015 to April 2021. This retrospective investigation was deemed exempt by the institutional review board of Stanford University, and patient consent was waived (IRB-64187).

### Patient Population

Patients of age18 years and above with baseline ASL imaging, seropositive LGI1 AE, negative Herpes Simplex Virus (HSV) PCR, negative Creuztfeld-Jakob Disease panel (4), absence of acute territorial infarct on MRI, and clinical presentation of stroke-like symptoms were included. Stroke-like symptoms included seizures, acute neurologic deficits, cognitive deficits, behavioral changes, altered mental status, memory loss, and myoclonus. This search yielded six patients with seropositive LGI1 AE included in our analysis. Four patients underwent follow-up MRI imaging, with ASL at least 3 weeks after the initial presentation.

### Imaging Acquisition and Post-processing

Patient examinations were performed from December 2015 to April 2021 on 3T Discovery (GE Healthcare, Milwaukee, Wisconsin) scanners.

ASL acquisition: ASL was performed using a single-delay pseudocontinuous (pCASL), with 3D eight-stacked spiral fast spin echo readout acquisition with background suppression, with the following imaging parameters: TR: 6512, TE: 10.2, FOV: 24 x 24 cm, slice thickness: 4 mm, scan time: 4 min, labeling duration: 1,500 ms, post-label delay: 2,000 ms, background suppressed three averages per acquisition based on the previously mentioned acquisition time. M0 scans were also acquired. ASL was reconstructed with an interpolated resolution of 1.9 × 1.9 × 4 mm^3^.

ASL post-processing: ASL post-processing was performed prior to CBF quantification for motion correction.

For generating CBF quantification maps, the previously described one compartment model for standard single delay ASL was utilized in this study (19, 20).

### Imaging Analysis

#### Qualitative Analyses

Imaging analysis of ASL CBF maps on the six patients with seropositive AE was performed independently by two neuroradiologists (VY, 3 years of experience; and BL, 6 years of experience) through qualitative visual inspection using a two-point scale (increased, decreased), comparing the abnormal signal to the unaffected ipsilateral and contralateral unaffected temporal and parietal cortices. Quality checks were performed by the two reading neuroradiologists to ensure the images were acceptable for diagnostic interpretation and free of a significant motion artifact or other confounding artifacts in the regions of interest, such as an arterial transit artifact. Discordant interpretations were resolved by consensus repeat review. The following characteristics were assessed: a) presence of ASL signal abnormality; b) if present, characterizing the ASL signal abnormality using a two-point scale, c) assessment of whether the ASL signal abnormality is in a territorial or non-territorial distribution, d) specific spatial anatomic location of the ASL signal abnormality, and e) laterality (left, right, or both; if both, which side predominantly was also specified).

#### Quantitative Analyses

Quantitative analysis was performed with manual placement of ROIs (placement performed by BL) in the region of the affected cortex in addition to the unaffected ipsilateral parietal, contralateral temporal, and contralateral parietal cortices. The ROI for the lesion was selected to cover only the abnormal mesial temporal lobe, but not the entirety of the abnormality. The contralateral side was measured at the same site and volume. For the unaffected brain, gray and white matter was selected, covering the same territory and volume on both sides. Abnormal CBF was defined as under 20 ml/min/100 g and above 80 ml/min/100 g (21).

## Results

In this study, we present six patients (five males and one female: 28–76 years of age, mean age, 55. years). Baseline characteristics are summarized in Table 1.

**Table 1:** Baseline characteristics.

**Baseline characteristic**	**Case 1**	**Case 2**	**Case 3**	**Case 4**	**Case 5**	**Case 6**
Age	70	28	57	54	48	76
Sex	M	M	M	F	M	M
Heart disease	Y	N	N	N	N	N/A
Hyperlipidemia	Y	N	Y	N	Y	N/A
Hypertension	Y	N	Y	Y	Y	N/A
Diabetes mellitus	N	N	Y	Y	N	N/A
Atrial fibrillation	N	N	Y	N	N	N/A
Smoking						
Non-smoker		Y	Y	Y	Y	
Former smoker	Y					Y
Current smoker						
Alcohol						
Non-alcoholic		Y		Y	Y	
Former alcoholic						
Current occasional consumer	Y		Y			Y
Current heavy consumer						
BMI	25.2	26.6	25	26.4	25.1	27.3

Four of the six underwent follow-up MRI, and three of the four had ASL available on follow-up imaging for review.

**Figure 1:**
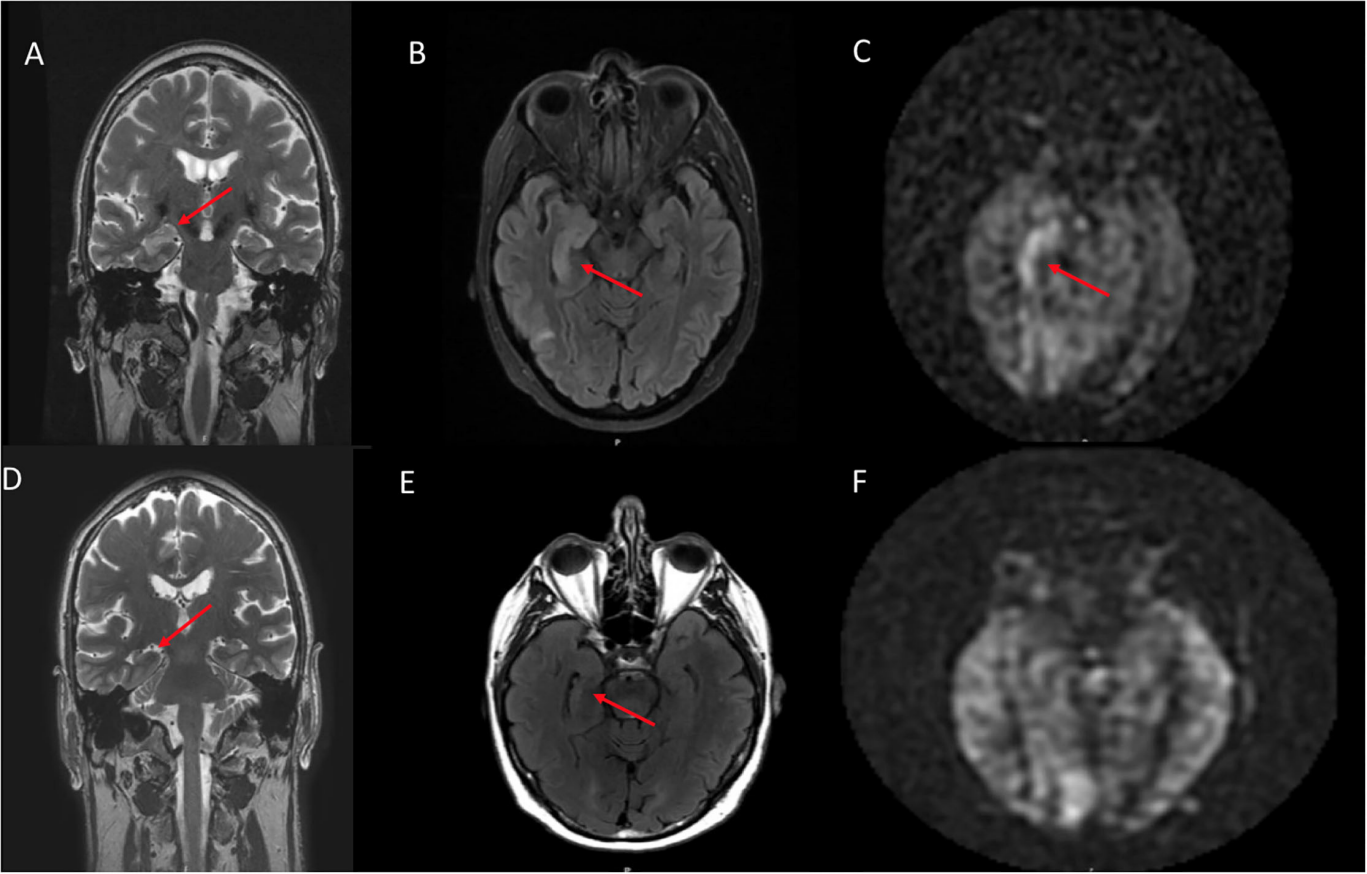
A 70 year old male who presented with rapid cognitive decline and spasmic extremity movement. **(A)** Initial coronal T2W shows asymmetric T2 hyperintense signal in the right hippocampus. **(B)** Axial FLAIR demonstrates asymmetric hyperintense signal in the right hippocampus and focal cortical signal abnormality in the posterior right temporal lobe. **(C)** ASL shows focal hyperperfusion in the right hippocampus and milder asymmetric signal in the posterior right temporal and right occipital lobes. **(D)** 12 month follow up coronal T2W shows mild right hippocampal volume loss. **(E)** Follow up axial FLAIR shows mild right hippocampal volume loss and hyperintense signal. **(F)** Follow up ASL shows complete resolution of the previously right hippocampal hyperperfusion. Of note, the decreased ASL signal in the left occipital lobe is due to the present of an ipsilateral fetal PCA.

**Figure 2:**
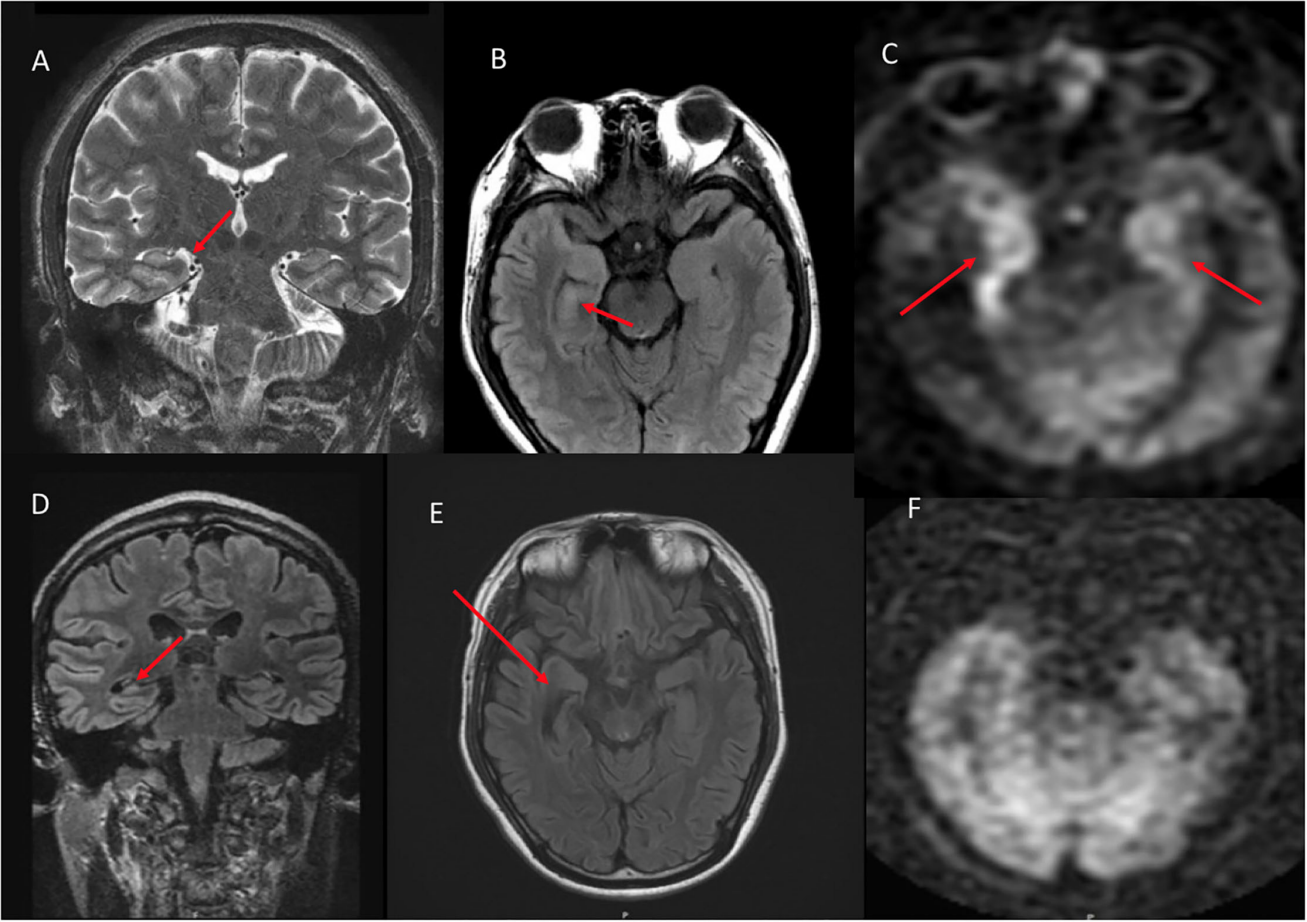
A 28 year old male who presented with seizures and altered mental status. **(A)** Coronal T2W shows mild right hippocampal edema. **(B)** Axial FLAIR shows subtle right hippocampal and amygadala hyperintense signal. **(C)** ASL demonstrates asymmetric right greater than left hyperperfusion. **(D)** 10 month follow up coronal FLAIR shows right hippocampal volume loss. **(E)** Axial FLAIR shows right hippocampal volume loss with associated ex vacuo dilatation of the right temporal horn. **(F)** ASL demonstrates resolution of the previous right greater than left hyperperfusion.

**Figure 3:**
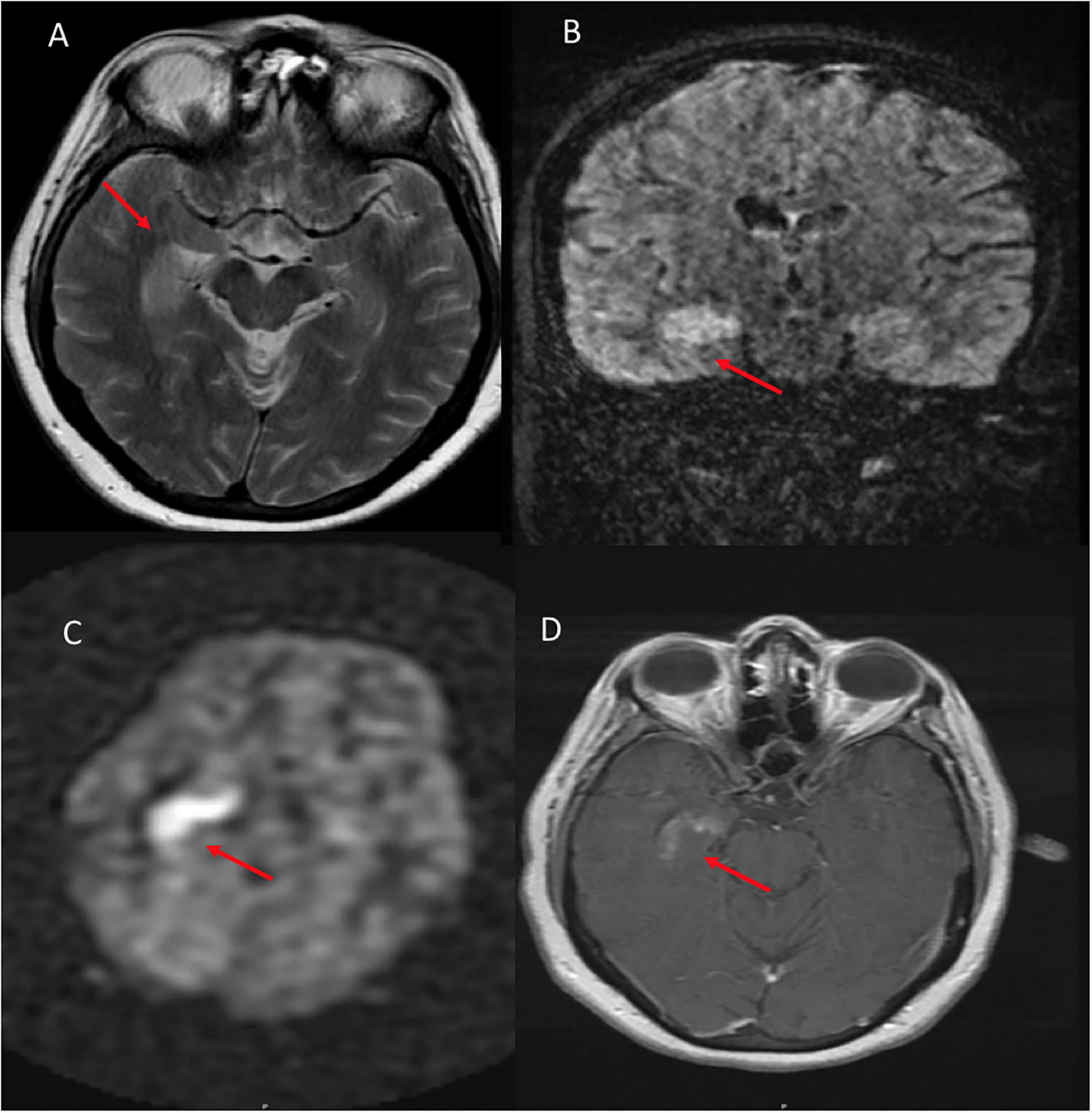
A 54 year old female who presented with altered mental status and seizures. **(A)** Axial T2W demonstrates right hippocampal hyperintense signal. **(B)** Coronal FLAIR shows corresponding hyperintense signal in the same region. **(C)** ASL shows hyperperfusion in the right hippocampus and amygdala. **(D)** T1W postcontrast shows heterogenous enhancement of the same region.

**Figure 4:**
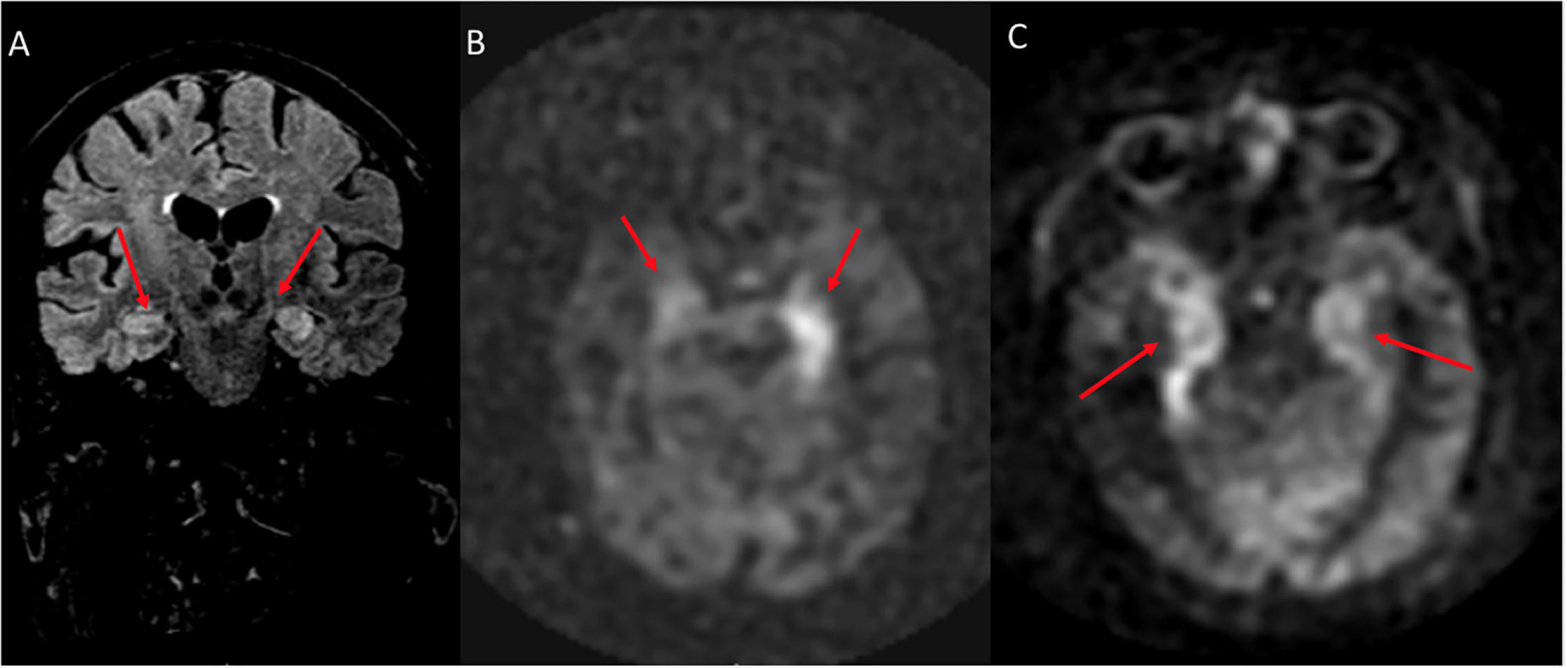
A 76 year old male with subacute cognitive decline. **(A)** Coronal FLAIR demonstrates hyperintense signal in the left greater than right hippocampus and amygdala. **(B)** ASL shows hyperperfusion in the left greater than right hippocampi. **(C)** ASL demonstrates bilateral involvement.

**Figure 5:**
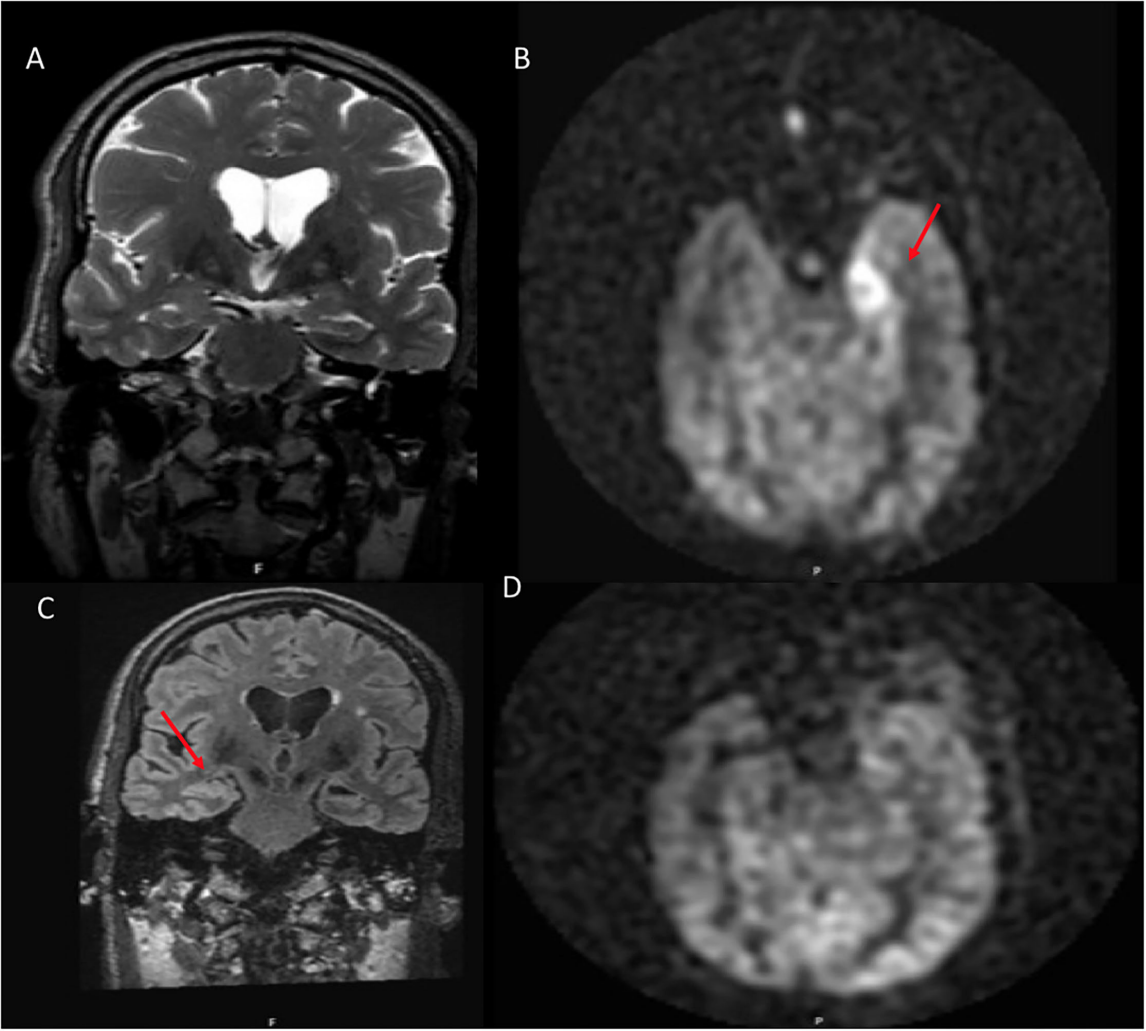
A 54 year old male with traumatic brain injury. **(A)** Coronal T2W shows no structural abnormality. **(B)** ASL demonstrates hyperperfusion within the left amygdala. **(C)** 8 month follow up coronal FLAIR shows left hippocampal volume loss. **(D)** ASL demonstrates resolution of the previous hyperperfusion.

**Figure 6:**
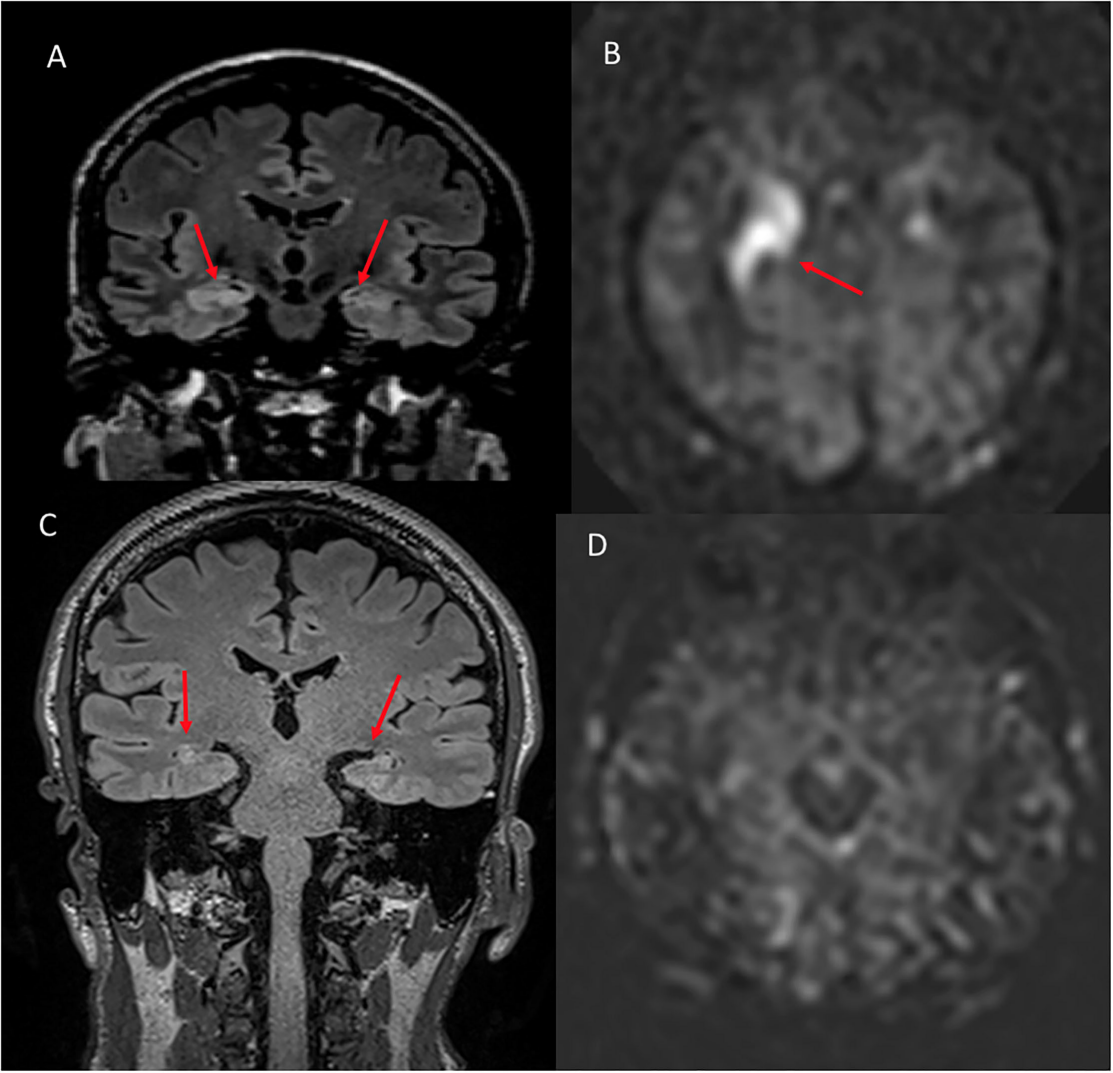
A 48 year old male with memory loss and seizures. **(A)** Coronal FLAIR shows hyperintense signal in the right greater than left hippocampus and amygdala. **(B)** ASL shows hyperperfusion in the corresponding regions. **(C)** Six month follow up coronal FLAIR shows hyperintense signal in the bilateral hippocampi compatible with mesial temporal sclerosis. **(D)** Six month follow up ASL demonstrates complete resolution of the previously seen hyperperfusion.

On qualitative consensus review, all patients had ASL signal abnormality present (6/6, 100%, Figures 1–4 on Panel B, as well as Figures 4–6 on Panel C). In all patients, the ASL signal abnormality was focally increased in the mesial temporal lobe involving the amygdala and hippocampus (5/6) or only the amygdala (1/6) in a non-territorial distribution. Associated T2/FLAIR hyperintensity and mild swelling were present in all cases (6/6 100%), although this finding was subtle in half of the cases. One patient had corresponding restricted diffusion (not shown) and enhancement in the affected mesial temporal cortex (1/6, 17%, Figure 3, Panel D). In five patients, the ASL signal abnormality involved the right mesial temporal lobe (5/6, 83%); one patient had left-sided mesial temporal lobe findings.

**Figure 7:**
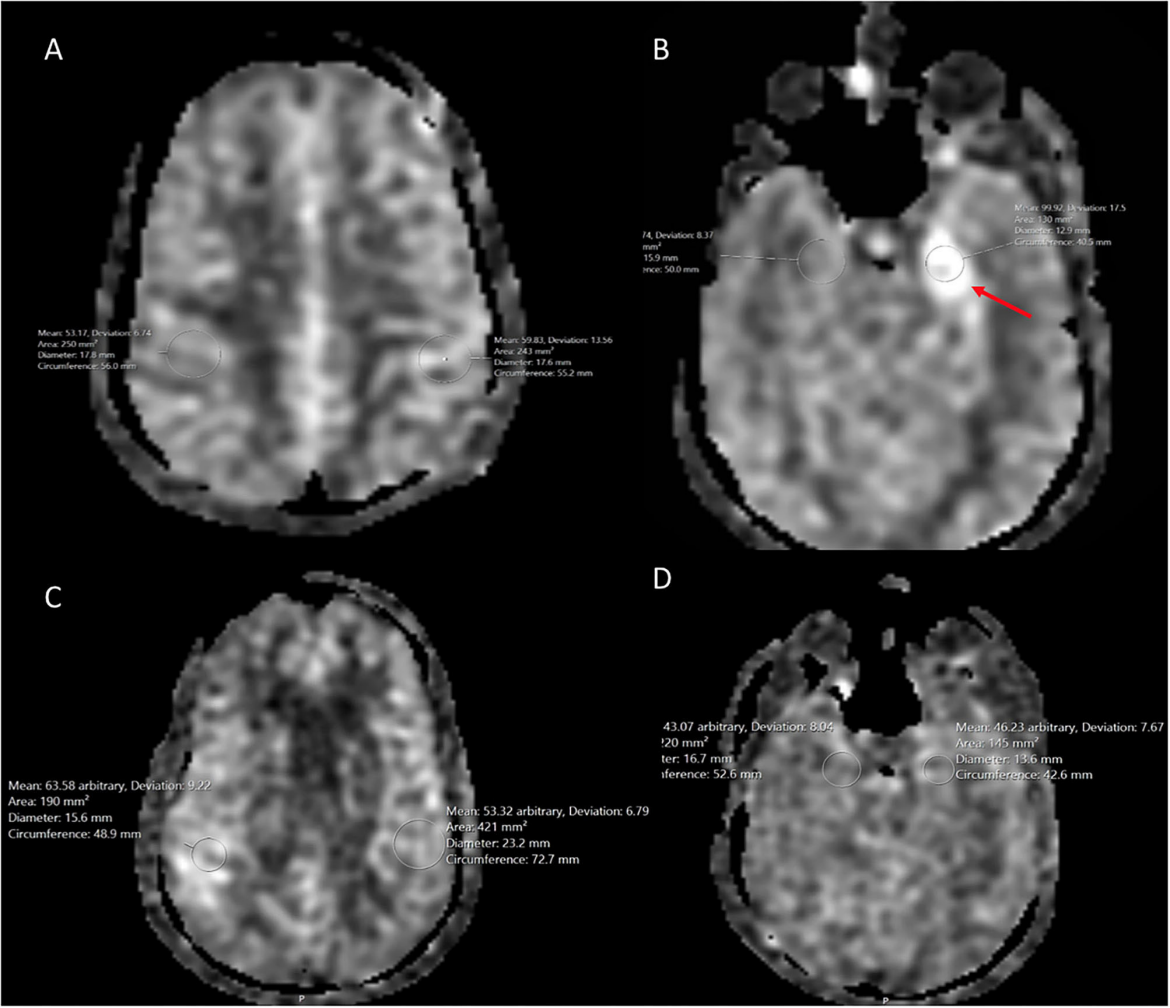
A 54 year old male with traumatic brain injury. **(A)** Initial ASL at the level of the parietal cortex shows normal cerebral blood flow bilaterally. **(B)** ASL demonstrates hyperperfusion within the left hippocampus when compared to the normal contralateral mesial temporal lobe. **(C)** 8 month follow up at the level of the parietal cortex demonstrates normal cerebral blood flow bilaterally. **(D)** 8 month follow up ASL shows resolution of the previous left hippocampal hyperperfusion with cerebral blood flow similar to the normal contralateral side.

On the four patients with ASL available on follow-up, the hyperperfusion was resolved in all patients (4/4, 100%, Figure 7 as an example). In three patients, the T2/FLAIR signal was resolved, while, in one patient, the edema seen on T2/FLAIR was resolved, but signal abnormality persisted in the same region of the ASL hyperperfusion, with development of volume loss when assessed qualitatively.

Quantitative analyses are summarized in Table 2.

**Table 2:** Quantitative ASL measurements by spatial location.

**Cerebral blood flow**	**Mean**	**Median**	**Standard deviation**	**Absolute difference between means of affected cortex and normal cortex per lobe**
CBF of affected temporal cortex	111.2 ± (5.73)	105 (100–131)	5.73	N/A
CBF of ipsilateral normal parietal cortex	49	49 (31–59)	N/A	62.2
CBF of contralateral normal temporal cortex	58.2 ± (5.62)	59 (44–77)	5.62	53
CBF of contralateral normal parietal cortex	52.2 ± (4.77)	52 (39–67)	4.77	59

Please see the detail sequence parameters in Table 3.

**Table 3:** Sequence parameters.

**Sequence**	**Readout**	**TR (ms)**	**TE (ms)**	**TI (ms)**	**Slice thickness (mm)**	**Acquisition matrix (voxels)**	**Flip angle**	**Acquisition time (mins)**
DWI (B 1000)	EPI	4,835	80.7		5	128 × 128	90	1:19
T2*W GRE	Gradient echo	680	15		5	384 × 224	20	2:18
3D FLAIR	Spin echo	6,002	136	1,709	1.2	256 × 256	90	5:03
2D FLAIR	Spin echo	9,500	147	2,300	5	384 × 224	111	2:48
3D T1W post contrast	Gradient echo	8.27	3.23	400	1	256 × 256	90	4:20
2DT1W post contrast	Spin echo	600	19		5	320 × 224	13	2:01

## Discussion

In this preliminary study of retrospectively reviewed patients, we demonstrate focal increased mesial temporal ASL signal abnormality compatible with hyperperfusion to be present with the LGI-1 subtype of AE on qualitative and quantitative analysis.

Prior studies have elucidated the structural MRI characteristics of AE and LGI1 in particular. In a retrospective review of eight patients with confirmed LGI1, Li et al. showed that, in five of the eight patients, there were T2 and FLAIR signal abnormalities involving the hippocampus, insula, or thalamus. In our study, we found mesial temporal involvement without signal abnormalities in the insula or thalamus. Kotsenas et al. and Kelley et al. both demonstrated that, in patients with voltage-gated potassium channels (VGKC) antibody AE - of which LGI1 is one of the most common (22), patients develop T2/FLAIR hyperintensity in the medial temporal lobes in the acute setting (2, 6). Also of note, Kotsenas et al. showed that patients with mesial temporal lobe FLAIR hyperintensity and associated restricted diffusion or postcontrast enhancement have a higher likelihood to progress to mesial temporal sclerosis (MTS) (6). Their findings are spatially concordant with our study on the mesial temporal lobe involvement. Flanagan et al. also reported that patients who present with FBDS in the setting of LGI1 also demonstrate T1 hyperintensity in the basal ganglia (23). Unlike Flanagan et al., we need not find any basal ganglia signal abnormality in any case.

Although structural MRI imaging has been increasingly investigated in AE and its subtypes, perfusion imaging is vastly underexplored and restricted only to case reports. Vallabhaneni et al. present one case of CT and MRI perfusion in a patient with GAD65 antibody AE, demonstrating hyperperfusion on both modalities in the left parieto-occipital cortex (17). Dinoto et al. reported ASL findings into two patients with LE - one seronegative and one LGI1. In the patient with LGI1, they found an increased ASL signal in the right hippocampus and the mesial temporal lobe along with FLAIR hyperintensity and postcontrast enhancement (18). Additionally, a case of an increased right mesial temporal lobe ASL signal within the hippocampus and amygdala has been previously reported in one patient with LGI1 (24).

Our preliminary analysis of six patients is the largest group of LGI1 patients with ASL reported to date. We report ASL hyperperfusion in a non-territorial distribution localized to the mesial cortex, specifically involving the hippocampal formation and/or amygdala, with a possible predilection for the right mesial temporal lobe. We postulate that the ASL hyperperfusion is found during the acute phase of the disease similar to what is seen with the ictal state in seizures (25). The distribution of the ASL hyperperfusion is concordant with prior investigations that have shown the LGI1 protein to be mainly expressed in the hippocampi and temporal cortex (26, 27).

Our findings are also in agreement with previous reports with the novel addition of ASL CBF quantification. Specifically, our finding of mesial temporal involvement falls in line with previous case reports by Dinoto et al. (18) and Espinosa-Jovel et al. (24). Interestingly, our finding is also concordant with previous reports of hypermetabolism in the right mesial temporal lobe on PET during the acute phase (7). This is also in contrast to the predominant cortical involvement seen in the patients with non-LGI1 and may represent a biomarker specific to LGI1. Given the high-relapse rate (4) and high mortality (28) of LGI1, it is also possible for ASL to be a potential treatment biomarker. In the four LGI1 patients with ASL follow-up imaging, all showed post-treatment resolution of the increased signal. However, despite subsequent resolution of the ASL hyperperfusion, all four patients were diagnosed with MTS. Larger-scale prospective studies are necessary to further investigate these findings.

Our study is not without limitations. We kept stringent criteria using a retrospective design that resulted in a small sample size of six patients with seropositive LGI-1 AE. Nevertheless, our cohort is the largest series of seropositive LGI1 patients with ASL reported in the literature. Notably, LGI1 is an exceedingly rare disease as LGI1 antibodies have only been recently discovered in 2010, and only 250 cases have been reported in the world as of 2015 (1). Only four of the six patients had follow-up imaging with ASL, limiting our temporal assessment post treatment. It is also notable that several patients presented acutely with seizures, which can also show an increased ASL signal in a non-territorial distribution in the ictal phase (11). It is certainly possible that the increased ASL signal in these patients is attributed to the ictal state. However, three of the six patients with LGI1 who did not present with seizures also had a similarly increased ASL signal in the right mesial temporal lobe, suggesting that this finding is distinct from the imaging findings seen in the ictal phase of seizures.

This study may serve as the basis for future investigations. Further larger scale retrospective or prospective studies with serial follow-up ASL imaging in this specific population are necessary to ascertain the strength of these findings.

## Conclusion

In this preliminary study, we qualitatively and quantitatively demonstrate an ASL hyperperfusion pattern with a possible predilection for the right mesial temporal lobe in patients with seropositive LGI1. Further studies must be performed to assess the strength of this potential association.

## Data Availability Statement

The raw data supporting the conclusions of this article will be made available by the authors, without undue reservation.

## Ethics Statement

Written informed consent was not obtained from the individual(s) for the publication of any potentially identifiable images or data included in this article.

## Author Contributions

VY: idea generation, primary writing, editing, and organization. OH: editing and organization. MB, VU, CJ, LL, and HS: editing. AA: reviewing, editing, and formatting. HL: reviewing and technical editing. BL: idea generation, editing, and organization. All authors contributed to the article and approved the submitted version.

## Funding

This paper was with the support of the Johns Hopkins University School of Medicine Department of Radiology Physician Scientist Incubator Program.

## Conflict of Interest

The authors declare that the research was conducted in the absence of any commercial or financial relationships that could be construed as a potential conflict of interest.

## Publisher's Note

All claims expressed in this article are solely those of the authors and do not necessarily represent those of their affiliated organizations, or those of the publisher, the editors and the reviewers. Any product that may be evaluated in this article, or claim that may be made by its manufacturer, is not guaranteed or endorsed by the publisher.

## References

[1] Thijs RD Coenders EC. Jiskoot LC Sanchez E de Bruijn MAAM. Anti-LGI1 encephalitis: Clinical syndrome and long-term follow-up. Neurology. (2016) 87:1449–56. 10.1212/WNL.0000000000003173 27590293

[2] Kelley BP Patel SC. Marin HL Corrigan JJ Mitsias PD Griffith B. Autoimmune encephalitis: pathophysiology and imaging review of an overlooked diagnosis. AJNR Am J Neuroradiol. (2017) 38:1070–8. 10.3174/ajnr.A5086 28183838 PMC7960083

[3] Dalmau J Graus F. Antibody-mediated encephalitis. N Engl J Med. (2018) 378: 840–51. 10.1056/NEJMra1708712 29490181

[4] Li W Wu S Meng Q Zhang X Guo Y Cong L. Clinical characteristics and short-term prognosis of LGI1 antibody encephalitis: a retrospective case study. BMC Neurol. (2018) 18:96. 10.1186/s12883-018-1099-z 29980179 PMC6035422

[5] Graus F Titulaer MJ Balu R Benseler S Bien CG Cellucci T. A clinical approach to diagnosis of autoimmune encephalitis. Lancet Neurol. (2016) 15: 391–404. 10.1016/S1474-4422(15)00401-9 26906964 PMC5066574

[6] Kotsenas AL Watson RE. Pittock SJ Britton JW Hoye SL Quek AML. MRI findings in autoimmune voltage-gated potassium channel complex encephalitis with seizures: one potential etiology for mesial temporal sclerosis. AJNR Am J Neuroradiol. (2014) 35:84–9. 10.3174/ajnr.A3633 23868165 PMC7966496

[7] Moreno-Ajona D Prieto E Grisanti F Esparragosa I Sánchez Orduz L Gállego Pérez-Larraya J. ^18^F-FDG-PET imaging patterns in autoimmune encephalitis: Impact of image analysis on the results. Diagnostics (Basel). (2020) 10:356. 10.3390/diagnostics10060356 32486044 PMC7344773

[8] Zaharchuk G. Arterial spin-labeled perfusion imaging in acute ischemic stroke. Stroke. Lippincott Williams and Wilkins. (2014) 7:1202–7. 10.1161/STROKEAHA.113.003612 24603069 PMC3967005

[9] El Mogy IS Fischbein NJ. Albers GW. Comparison of arterial spin labeling and bolus perfusion-weighted imaging for detecting mismatch in acute stroke. Stroke. (2012) 43:1843–8. 10.1161/STROKEAHA.111.639773 22539548 PMC3383868

[10] Yedavalli V Tong E. The potential utility of arterial spin labeling in detecting and localizing posterior circulation occlusions in every day practice: a clinical report of selected cases. J Clin Imaging Sci. (2020) 10:78. 10.25259/JCIS_118_2020 33365200 PMC7749935

[11] Telischak NA Detre JA Zaharchuk G. Arterial spin labeling MRI: clinical applications in the brain. J Magn Reson Imaging. (2015) 41:1165–80. 10.1002/jmri.24751 25236477

[12] Haller S Zaharchuk G Thomas DL Lovblad K-O Barkhof F Golay X. Arterial spin labeling perfusion of the brain: Emerging clinical applications. Radiology. (2016) 281:337–56. 10.1148/radiol.2016150789 27755938

[13] Yedavalli V Lanzman B. A potential new role for ASL perfusion imaging: Diagnosis of metronidazole induced encephalopathy – Two companion cases. Radiol Case Rep. (2020) 15:11. 10.1016/j.radcr.2019.10.011 31737151 PMC6849432

[14] Detre JA Zager EL Hurst RW. Arteriovenous shunt visualization in arteriovenous malformations with arterial spin-labeling MR imaging. AJNR Am J Neuroradiol. (2008) 29:681–7. 10.3174/ajnr.A0901 18397967 PMC7978181

[15] Wang Y-H An Y Fan X-T Lu J Ren L-K Wei P-H. Comparison between simultaneously acquired arterial spin labeling and ^18^F-FDG PET in mesial temporal lobe epilepsy assisted by a PET/MR system and SEEG. Neuroimage Clin. (2018) 19:824–30. 10.1016/j.nicl.2018.06.008 30013926 PMC6024198

[16] Sachs JR Zapadka ME Popli GS Burdette JH. Arterial spin labeling perfusion imaging demonstrates cerebral hyperperfusion in anti-NMDAR encephalitis. Radiol Case Rep. (2017) 12:833–7. 10.1016/j.radcr.2017.06.004 29484082 PMC5823289

[17] Vallabhaneni D Naveed MA Mangla R Zidan A Mehta RI. Perfusion imaging in autoimmune encephalitis. Case Rep Radiol. (2018) 2018: 3538645. 10.1155/2018/3538645 29854534 PMC5960559

[18] Dinoto A Cheli M Ajčević M Dore F Crisafulli C Ukmar M. ASL MRI and ^18^F-FDG-PET in autoimmune limbic encephalitis: clues from two paradigmatic cases. Neurol Sci. (2021) 42: 3423–5. 10.1007/s10072-021-05207-0 33763811

[19] Fan AP Khalighi MM Guo J Ishii Y Rosenberg J Wardak M. Identifying hypoperfusion in moyamoya disease with arterial spin labeling and an [^15^O]-water positron emission tomography/magnetic resonance imaging normative database. Stroke. (2019) 50:373–80. 10.1161/STROKEAHA.118.023426 30636572 PMC7161423

[20] Alsop DC Detre JA Golay X Günther M Hendrikse J Hernandez-Garcia L. Recommended implementation of arterial spin-labeled perfusion MRI for clinical applications: A consensus of the ISMRM perfusion study group and the European consortium for ASL in dementia. Magn Reson Med. (2015) 73:102–16. 10.1002/mrm.25197 24715426 PMC4190138

[21] Fantini S Sassaroli A Tgavalekos KT Kornbluth J. Cerebral blood flow and autoregulation: current measurement techniques and prospects for noninvasive optical methods. Neurophotonics. (2016) 3:031411. 10.1117/1.NPh.3.3.031411 27403447 PMC4914489

[22] Celicanin M Blaabjerg M Maersk-Moller C Beniczky S Marner L Thomsen C. Autoimmune encephalitis associated with voltage-gated potassium channels-complex and leucine-rich glioma-inactivated 1 antibodies - a national cohort study. Eur J Neurol. (2017) 24:999–1005. 10.1111/ene.13324 28544133

[23] Flanagan EP Kotsenas AL. Britton JW McKeon A Watson RE Klein CJ. Basal ganglia T1 hyperintensity in LGI1-autoantibody faciobrachial dystonic seizures. Neurol Neuroimmunol Neuroinflamm. (2015) 2:e161. 10.1212/NXI.0000000000000161 26468474 PMC4592539

[24] Espinosa-Jovel C Toledano R García-Morales I Álvarez-Linera J Gil-Nagel A. Serial arterial spin labeling MRI in autonomic status epilepticus due to anti-LGI1 encephalitis. Neurology. (2016) 87: 443–4. 10.1212/WNL.0000000000002903 27462038

[25] Yoo RE Yun TJ Yoon BW Lee SK Lee ST Kang KM. Identification of cerebral perfusion using arterial spin labeling in patients with seizures in acute settings. PLoS ONE. (2017) 12:3538. 10.1371/journal.pone.0173538 28291816 PMC5349669

[26] Shao X Fan S Luo H Wong TY Zhang W Guan H. Brain magnetic resonance imaging characteristics of anti-leucine-rich glioma-inactivated 1 encephalitis and their clinical relevance: a single-center study in China. Front Neurol. (2021) 11:618109. 10.3389/fneur.2020.618109 33510707 PMC7835512

[27] Herranz-Pérez V Olucha-Bordonau FE Morante-Redolat JM Pérez-Tur J. Regional distribution of the leucine-rich glioma inactivated (LGI) gene family transcripts in the adult mouse brain. Brain Res. (2010) 1307:177–94. 10.1016/j.brainres.2009.10.013 19833108

[28] Ariño H Armangué T Petit-Pedrol M Sabater L Martinez-Hernandez E Hara M. Anti-LGI1-associated cognitive impairment: Presentation and long-term outcome. Neurology. (2016) 87: 759–765. 10.1212/WNL.0000000000003009 27466467 PMC4999321

